# Secondary S100B Protein Increase Following Brain Arteriovenous Malformation Rupture is Associated with Cerebral Infarction

**DOI:** 10.3390/molecules25215177

**Published:** 2020-11-06

**Authors:** Lorenzo Garzelli, Alice Jacquens, Caroline Amouyal, Kevin Premat, Nader Sourour, Jonathan Cortese, Idriss Haffaf, Bertrand Mathon, Stéphanie Lenck, Frédéric Clarençon, Vincent Degos, Eimad Shotar

**Affiliations:** 1 Department of Neuroradiology, Pitié-Salpêtrière Hospital, 75013 Paris, France; lorenzo.garzelli@aphp.fr (L.G.); kevin.premat@aphp.fr (K.P.); nsourour@gmail.com (N.S.); jon.cortese@live.fr (J.C.); ydriss92@hotmail.fr (I.H.); stephanie.lenck@aphp.fr (S.L.); frederic.clarencon@aphp.fr (F.C.); 2 Neurosurgical Anesthesiology and Critical Care, Pitié-Salpêtrière Hospital, 75013 Paris, France; alice.jacquens@gmail.com (A.J.); caroline.amouyal@gmail.com (C.A.); vincent.degos@aphp.fr (V.D.); 3 Medical Faculty, Sorbonne Université, 75013 Paris, France; bertrand.mathon@aphp.fr; 4 Department of Neurosurgery, Pitié-Salpêtrière Hospital, 75013 Paris, France

**Keywords:** cerebrovascular malformations, intracranial hemorrhage, prognosis, arteriovenous malformations, brain, stroke, biomarker

## Abstract

Early S100B protein serum elevation is associated with poor prognosis in patients with ruptured brain arteriovenous malformations (BAVM). The purpose of this study is to determine whether a secondary elevation of S100B is associated with early complications or poor outcome in this population. This is a retrospective study of patients admitted for BAVM rupture. A secondary increase of S100B was defined as an absolute increase by 0.1 μg/L within 30 days of admission. Fisher’s and unpaired t tests followed by multivariate analysis were performed to identify markers associated with this increase. Two hundred and twenty-one ruptures met inclusion criteria. Secondary S100B protein serum elevation was found in 17.1% of ruptures and was associated with secondary infarction (*p* < 0.001), vasospasm-related infarction (*p* < 0.001), intensive care (*p* = 0.009), and hospital length of stay (*p* = 0.005), but not with early rebleeding (*p* = 0.07) or in-hospital mortality (*p* = 0.99). Secondary infarction was the only independent predictor of secondary increase of S100B (OR 9.9; 95% CI (3–35); *p* < 0.001). Secondary elevation of S100B protein serum levels is associated with secondary infarction in ruptured brain arteriovenous malformations.

## 1. Introduction

Brain arteriovenous malformation (BAVM) rupture results in significant morbi-mortality, but the prognosis is better than primary hematoma [1]. Elevated S100B serum levels within the first 48 h of patient admission are found to be an independent predictor of in-hospital mortality following BAVM rupture [2]. Furthermore, a secondary increase of S100B is described as a significant marker of severe neurological complications in routinely admitted neuro-intensive care patients [3]. Secondary complications following BAVM rupture include hydrocephalus, rebleeding, and vasospasm [4,5,6]. No study to this date has monitored S100B in ruptured BAVM beyond 48 h. The present study aims to describe S100B evolution and determine whether a secondary elevation is associated with early complications or poorer outcomes following BAVM rupture.

## 2. Results

### 2.1. Population

**Figure 1:**
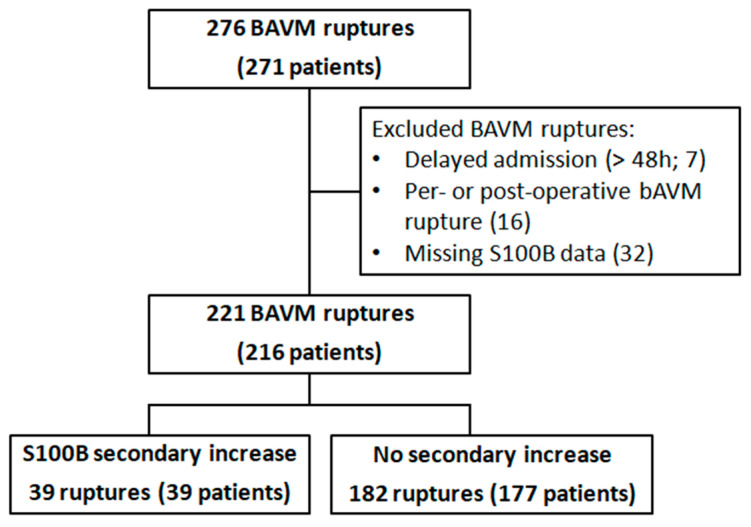
Flow-chart.

Two hundred and seventy-one patients with 276 ruptures were identified during the study period. A total of 221 hemorrhagic events in 216 patients were included for analysis (Figure 1). Sixteen patients (7%) were tachycardic and 6 patients (3%) were bradycardic at admission. Fifty-seven patients (26%) and 7 patients (3%) had, respectively, a high and a low mean blood pressure at admission. Eighty-six percent of the patients had normal levels of troponin. Patients’ characteristics are displayed in Table 1.

### 2.2. S100B Evolution in General Population

**Figure 2:**
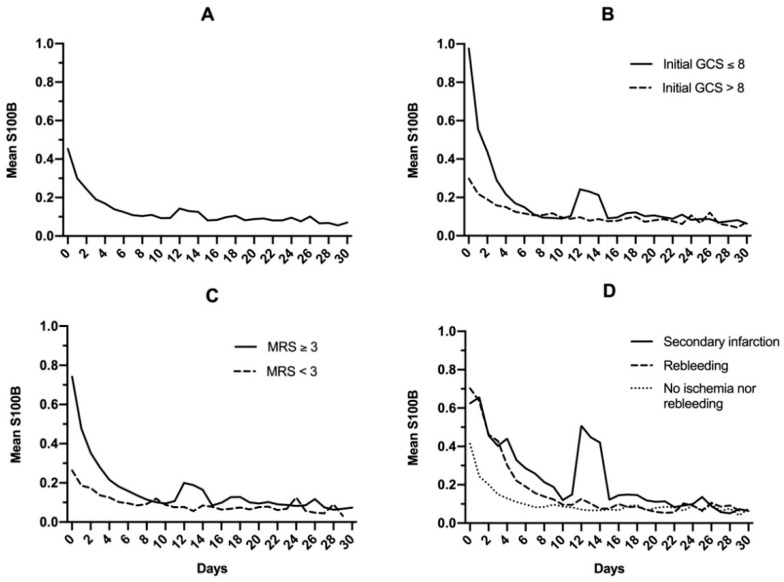
Mean S100B protein serum level in the general population (**A**), after dichotomizing based on initial Glasgow coma scale (GCS) (**B**), modified Rankin Scale (**C**), and late complications following brain arteriovenous malformation rupture (**D**).

**Figure 3:**
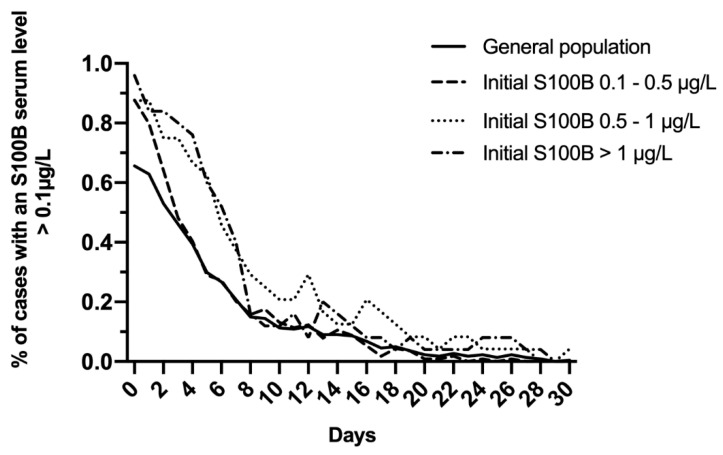
Evolution of the proportion of cases with S100B serum level >0.1 μg/L in the total study population and after dichotomizing on initial S100B serum level (0.1–0.5 μg/L; 0.5–1 μg/L; >1 μg/L).

In the total study population, S100B decreased daily to reach normal levels within 10 days (Figure 2A). A modest increase of mean levels was observed on day 12, with a mean S100B level of 0.14 μg/L, and this increase was prolonged up to day 14 (0.12 μg/L). Whatever the initial S100B serum level (total study population; 0.1–0.5 μg/L; 0.5–1 μg/L; >1 μg/L), more than 80% of patients reached normal S100B serum levels within the first 7 to 10 days following admission (Figure 3).

### 2.3. S100B Evolution in Subgroups

When the general population was dichotomized based on initial clinical Glasgow coma scale (GCS) (either ≤8 or >8; Figure 2B) or outcomes (modified Rankin Scale <3 or ≥3; Figure 2C), a secondary increase in S100B levels was noted for patients with GCS ≤8 (0.10 to 0.24 μg/L on day 12) and modified Rankin Scale ≥3 (0.10 to 0.19 μg/L on day 12). When mean S100B levels were then dichotomized based on secondary complications (infarction or early rebleeding; Figure 2D), secondary increase was found to be exclusively related to infarction. In this subgroup, a notable increase in mean S100B levels was observed, rising from 0.12 μg/L on day 10 to 0.50 μg/L on day 12. This secondary increase did not prevent S100B from reaching normal levels on day 30 (0.06 μg/L). Causes and description of cerebral infarctions are displayed in Table 2.

### 2.4. Exploratory Analysis

Exploratory analysis was performed to identify variables associated with secondary S100B protein serum elevation (Table 3). When multivariate logistic regression was performed, the only independent predictor of secondary S100B increase was secondary infarction (OR 9.9; 95% CI (3–35); *p* < 0.001). As for outcome, secondary increase of S100B protein was associated with a duration of stay in intensive care ≥ 1 month (*p* = 0.009) and a longer hospitalization (*p* = 0.005). A trend towards increased sedation times was found without reaching significance (*p* = 0.06).

## 3. Discussion

In this large population with ruptured BAVM, secondary S100B protein serum elevation was associated with ischemic complications, particularly vasospasm-related infarction and a prolonged stay both in the intensive care unit and hospital.

Markers of initial severity following BAVM rupture as well as long-term outcome have been widely studied [1,7,8,9]. In contrast, studies evaluating the impact of early complications (and their incidence) such as rebleeding or ischemia remain scarce [5,6,10].

Despite modern techniques of neuromonitoring, prompt diagnosis and correction of complications represents a challenge when providing care for comatose and/or sedated patients. In some cases, differentiating primary disease aggravation from the onset of a complication can prove to be challenging. In this setting, early and extended S100B protein monitoring has been found to be a reliable, non-invasive, and accessible marker [3].

Elevated S100B serum within 48 h following admission has been found to be an independent predictor of in-hospital mortality in ruptured BAVM, stronger than imaging markers [2]. The present study provides an extended S100B protein levels analysis with data obtained within the first month of admission.

Secondary infarction was responsible for a secondary increase of S100B protein. Regarding the infarction rate reported herein, a previous study by Chhor et al. [6] found a similar proportion of 8% of cerebral infarction (all caused by vasospasm) and 17% of vasospasm (non-accountable for infarction) in 72 ruptured BAVM. Prolonged intensive care unit and hospitalization stay, in a patient with secondary increase of S100B serum protein level, could be attributed to secondary infarction. In this study, early rebleeding failed to reach statistical significance in its association with S100B secondary increase. A previous study [5] found that early rebleeding was associated with poor neurological outcome but not with in-hospital mortality, and could suggest that rebleeding may not be responsible for sufficient brain cell damage to release S100B protein in the serum, unlike infarction. The low rate of secondary infarction could underpower its effect on in-hospital mortality and thus explain the lack of significance.

The monocentric retrospective design of this study is a source of bias. Most data regarding S100B on the specific population with ruptured BAVM and its early complications originate from our center, limiting external validity. The impact of vasospasm without infarction on S100B secondary increase was not evaluated but is unlikely to exist. Heart participation in the release of S100B protein should also be discussed. It has been proven that S100B protein can be released from the ischemic heart [11,12]. S100B has also been found to be an independent prognosis factor in chronic heart failure and major cardiac events [13]. Cardiac participation in the secondary increase of S100B should be questioned. In this study there was no significant difference in troponin levels between the two groups at admission, despite known positive correlation between the two proteins [14]. However, troponin during hospitalization was not monitored. It is therefore not possible to exclude a joint release of S100B by the brain and the heart, however unlikely. Even so, these results demonstrate that S100B secondary elevation was likely to be related to cerebral infarction, and the putative origin of its release does not undermine this specific result. Finally, discontinued sampling of S100B protein in some cases, after several days of normal levels, is also a limitation, stressing the need for prospective studies with continued measurement of patients with ruptured BAVM.

## 4. Materials and Methods

### 4.1. Patients

Records of adult patients (age >15 years old) with BAVM ruptures, admitted to a tertiary care teaching center between 1 January 2003, and 1 February 2020, were retrospectively reviewed. One hundred and ninety-two ruptures included in this study were previously included in a report of early S100B protein levels after BAVM ruptures [2]. Demographic, clinical, biological, and imaging characteristics of patients were reviewed. At admission, patients’ troponin levels were recorded (troponin I before June 2013 and Hs thereafter). Outcomes analyzed included in-hospital mortality, poor neurological outcome (defined by a Modified Rankin Scale ≥3 at least 3 months after admission), length of stay, sedation time, external ventricular drainage, early obliteration (surgical and/or embolization within 1 month), early rebleeding (within 1 month of an untreated BAVM), and the occurrence of a secondary infarction from any cause during hospitalization. Infarction was defined on magnetic resonance imaging (MRI) as a hypersignal in a diffusion-weighted imaging sequence associated with a restricted apparent diffusion coefficient. On computed tomography, infarction was defined as a cortical and subcortical hypo-attenuated area with an arterial vascular distribution associated with a vascular spasm or occlusion. Silent microemboli on MRI were excluded as they can be relayed with diagnostic cerebral angiography [15].

### 4.2. Ethical Statement

The ethics committee of our institution approved this study. The need for patients’ informed consent was waived by the Institutional Review Board.

### 4.3. Statistical Analysis

Continuous variables were expressed as means (standard deviations) or medians (interquartile range) as appropriate. Categorical variables were expressed as absolute numbers with percentages. Fisher’s exact test or unpaired t-test with Welch’s correction were performed when appropriate. Multiple logistic regression analysis was performed to identify predictors of secondary S100B protein elevation. Variables found to be predictive in univariate analysis with *p* values <0.1 were included. Early rebleeding was forced into the model because of clinical significance. *p* values <0.05 were considered significant. Statistical analysis was performed using Prism version 8.3.1 (GraphPad Software, San Diego, CA, USA).

### 4.4. S100B Protein Serum Level

S100B serum levels at admission and subsequent sampling were retrospectively reviewed. In most patients, samples were drawn on admission and daily afterwards. Some patients had several samples drawn on the same day (because of acute deterioration for instance), and only the first was used for analysis. In some cases, mostly after several days of normal S100B levels, sampling was discontinued until occurrence of neurological deterioration. Normal S100B level was arbitrarily defined as <0.1 μg/L in this study. A secondary increase of S100B serum level was defined as an absolute elevation of 0.1 μg/L at any point in time. Analysis was stopped at 30 days after admission. Physicians were aware of S100B serum level values in real time. Since December 2, 2010, S100B has been assayed on ModularE170 analyzer (Roche, Mannheim, Germany). Results assessed before that date were measured on a LiaisonXL (Diasorin, Saluggia, Italy); therefore, these values were corrected as (concentration − 0.01)/2.28, according to the linear regression between the two methods as previously reported [16].

## 5. Conclusions

Secondary elevation of S100B protein serum levels is associated with secondary infarction and prolonged hospitalization in patients with ruptured BAVM. Whether monitoring S100B protein serum levels could help diagnose secondary infarctions, especially in deep comatose patients, should be evaluated.

## Figures and Tables

**Table 1:** Demographic, clinical, biological, and imaging characteristics of patients presenting with brain arteriovenous malformation (BAVM) ruptures.

	221 BAVM Ruptures (216 Patients)
**Demographics and Medical History**	
Age	44 ± 15
Male	124 (56)
History of high blood pressure (*n* = 220)	34 (15)
Past BAVM rupture (*n* = 220)	23 (10)
**Admission Characteristics**	
Median initial GCS (*n* = 218)	14 (10–15)
Initial GCS ≤ 8	49 (22)
Heart rate (*n* = 191)	75 ± 16
Mean blood pressure (*n* = 197)	99 ± 18
**BAVM Characteristics**	
Nidus size (*n* = 207)	22 ± 16
Exclusive deep venous drainage (*n* = 199)	55 (28)
Deep localization (*n* = 217)	48 (22)
Any aneurysm (*n* = 197)	91 (46)
Nidal aneurysm (*n* = 196)	53 (27)
Flow-related aneurysm (*n* = 207)	53 (26)
Median Spetzler-Martin grade (*n* = 204)	2
1–2	125 (61)
3	55 (27)
4–5	24 (12)
**Hemorrhage Characteristics**	
Infratentorial cerebral hemorrhage (*n* = 219)	37 (17)
Intracerebral hemorrhage ≥30 mL (*n* = 217)	97 (45)
Intracerebral hemorrhage ≥60 mL (*n* = 217)	50 (23)
Intraventricular hemorrhage (*n* = 219)	143 (65)
Subarachnoid hemorrhage (*n* = 219)	54 (25)
Subdural hematoma (*n* = 219)	18 (8)
**Biological Parameters**	
Mean Initial S100B (*n* = 215)	0.44 ± 0.60
Mean S100B on day 3 (*n* = 166)	0.19 ± 0.23
Mean S100B on day 7 (*n* = 132)	0.10 ± 0.12
Mean S100B at 1 month (*n* = 4)	0.07 ± 0.04
Plasma troponin elevation (*n* = 169)	24 (14)
Serum creatinine (µmol/L) (*n* = 203)	63 ± 19
**Management and Outcome**	
EVD placement	122 (55)
Early obliteration (<1 month)	58 (26)
Early rebleeding (<1 month)	14 (6)
Secondary infarction	18 (8)
Vasospasm-related infarction	5 (2)
Intra-hospital mortality	25 (11)
Mean intensive care length of stay * (*n* = 164)	30 ± 47
Mean hospital length of stay * (*n* = 151)	38 ± 35
Mean sedation time (days)	4.30 ± 8.5
Modified Rankin Scale ≥3 ^†^ (*n* = 214)	87 (41)

Values are mean ± standard deviation or medians with interquartile range for quantitative variables and percentage for qualitative variables. * In-hospital deaths are not counted. ^†^ Beyond 3 months. Abbreviations: BAVM (brain arteriovenous malformation); EVD (external ventricular drainage); GCS (Glasgow coma scale).

**Table 2:** Causes of cerebral infarction complicating brain arteriovenous malformation ruptures.

Etiology	Number of Ruptures (%)	Mean Delay from Admission (Days; Range)	Ischemic Territories	Diagnosis Modality	Comments
Vasospasm	5 (28%) *	8 (4–14)	ACA = 2 MCA = 4 PCA = 1 VB = 3	MRI = 2 CT = 3	
Iatrogenic	7 (39%) ^†^	9.2 (0–42)	ACA = 1 MCA = 5 PCA = 1 VB = 2	MRI = 3 CT = 4	Embolization: 4 Hematoma evacuation: 3
Mass effect	5 (28%)	1.8 (0–4)	ACA = 2 PCA = 2 VB = 1	MRI = 2 CT = 3	Brain herniation: 2 Compressive hematoma: 3
Sporadic	1 (5%)	6	PCA	CT	Patent foramen ovale
Unknown	1 (5%)	1	ACA	CT	

One patient had both iatrogenic and vasospasm-related infarction. * Three patients had more than one territory involved. ^†^ Two patients had more than one territory involved. Abbreviations: ACA (anterior cerebral artery); CT (computed tomography); MCA (middle cerebral artery); MRI (magnetic resonance imaging); PCA (posterior cerebral artery); VB (vertebrobasilar).

**Table 3:** Variables associated with S100B secondary increase.

	S100B Secondary Increase (39/221 BAVM Ruptures)	No Secondary Increase (182/221 BAVM Ruptures)	Univariate Analysis of Predictive Factors for S100B Secondary Increase
			*p* Value
**Demographics and Medical History**			
Age (years)	48 ± 15	42 ± 15	0.058
Male	22/39 (56)	102/182 (56)	0.96
History of high blood pressure	8/39 (20)	26/181 (14)	0.33
Past BAVM rupture	4/39 (10)	19/181 (10)	0.99
**Admission Characteristics**			
Median initial GCS	14 (10–15)	14 (10–15)	0.83
Initial GCS ≤8	9/39 (23)	40/179 (22)	0.99
Heart rate (bpm)	73 ± 18	75 ± 15	0.65
Mean blood pressure (mmHg)	107 ± 25	97 ± 16	0.038 *^,†^
Plasma troponin elevation	5/31 (16)	19/138 (14)	0.77
Serum creatinine (µmol/L)	62 ± 21	64 ± 19	0.72
**Hemorrhage Characteristics**			
Infratentorial cerebral hemorrhage	9/39 (23)	28/180 (15)	0.24
Intracerebral hemorrhage ≥30 mL	13/39 (33)	84/178 (47)	0.15
Intraventricular hemorrhage	30/39 (76)	113/180 (62)	0.09
Subarachnoid hemorrhage	13/39 (33)	41/180 (22)	0.21
Subdural hematoma	1/39 (2)	17/180 (9)	0.20
**Management and Outcome**			
EVD placement	30/39 (76)	92/182 (50)	0.002 *^,†^
Early obliteration (<1 month)	10/39 (25)	48/182 (26)	0.99
Early rebleeding (<1 month)	5/39 (12)	9/182 (4)	0.07 *
Secondary infarction	12/39 (30)	6/180 (3)	<0.001 *^,†^
Vasospasm-related infarction	5/32 (15)	0/174 (0)	<0.001 ^†^
Intensive care length of stay ≥30 days	19/34 (55)	40/130 (30)	0.009 ^†^
Mean hospital length of stay (days)	62 ± 53	32 ± 26	0.005 ^†^
Mean sedation time (days)	7 ± 8	3 ± 8	0.06
Modified Rankin Scale ≥3	18/38 (47)	69/176 (39)	0.36
Intra-hospital mortality	4/39 (10)	21/182 (11)	0.99

Values are mean ± standard deviation or medians with interquartile range for quantitative variables and percentages for qualitative variables. Abbreviations: BAVM (brain arteriovenous malformation); bpm (beats per minute); GCS (Glasgow coma scale). * Indicates variables included in the multivariate analysis. ^†^ Statistical significance (*p* < 0.05).

## References

[1] Choi J.H., Mast H., Sciacca R.R., Hartmann A., Khaw A.V., Mohr J.P., Sacco R.L., Stapf C. (2006). Clinical outcome after first and recurrent hemorrhage in patients with untreated brain arteriovenous malformation. Stroke.

[2] Shotar E., Amouyal C., Jacquens A., Mathon B., Boulouis G., Monneret D., Premat K., Lenck S., Sourour N.A., Clarencon F. (2019). S100B Serum Elevation Predicts In-Hospital Mortality After Brain Arteriovenous Malformation Rupture. Stroke.

[3] Raabe A., Kopetsch O., Woszczyk A., Lang J., Gerlach R., Zimmermann M., Seifert V. (2004). S-100B protein as a serum marker of secondary neurological complications in neurocritical care patients. Neurol. Res..

[4] Gross B.A., Lai P.M., Du R. (2013). Hydrocephalus after arteriovenous malformation rupture. Neurosurg. Focus.

[5] Shotar E., Pistocchi S., Haffaf I., Bartolini B., Jacquens A., Nouet A., Chiras J., Degos V., Sourour N.A., Clarencon F. (2017). Early Rebleeding after Brain Arteriovenous Malformation Rupture, Clinical Impact and Predictive Factors: A Monocentric Retrospective Cohort Study. Cerebrovasc. Dis..

[6] Chhor V., Le Manach Y., Clarencon F., Nouet A., Daban J.L., Abdennour L., Puybasset L., Lescot T. (2011). Admission risk factors for cerebral vasospasm in ruptured brain arteriovenous malformations: An observational study. Crit. Care.

[7] Shotar E., Debarre M., Sourour N.A., Di Maria F., Gabrieli J., Nouet A., Chiras J., Degos V., Clarencon F. (2018). Retrospective study of long-term outcome after brain arteriovenous malformation rupture: The RAP score. J. Neurosurg..

[8] van Beijnum J., Lovelock C.E., Cordonnier C., Rothwell P.M., Klijn C.J., Al-Shahi Salman R. (2009). Outcome after spontaneous and arteriovenous malformation-related intracerebral haemorrhage: Population-based studies. Brain.

[9] Murthy S.B., Merkler A.E., Omran S.S., Gialdini G., Gusdon A., Hartley B., Roh D., Mangat H.S., Iadecola C., Navi B.B. (2017). Outcomes after intracerebral hemorrhage from arteriovenous malformations. Neurology.

[10] Gross B.A., Du R. (2012). Vasospasm after arteriovenous malformation rupture. World Neurosurg..

[11] Mazzini G.S., Schaf D.V., Oliveira A.R., Gonçalves C.A., Belló-Klein A., Bordignon S., Bruch R.S., Campos G.F., Vassallo D.V., Souza D.O. (2005). The ischemic rat heart releases S100B. Life Sci..

[12] Cai X.Y., Lu L., Wang Y.N., Jin C., Zhang R.Y., Zhang Q., Chen Q.J., Shen W.F. (2011). Association of increased S100B, S100A6 and S100P in serum levels with acute coronary syndrome and also with the severity of myocardial infarction in cardiac tissue of rat models with ischemia-reperfusion injury. Atherosclerosis.

[13] Li J.P., Lu L., Wang L.J., Zhang F.R., Shen W.F. (2011). Increased serum levels of S100B are related to the severity of cardiac dysfunction, renal insufficiency and major cardiac events in patients with chronic heart failure. Clin. Biochem..

[14] Csecsei P., Pusch G., Ezer E., Berki T., Szapary L., Illes Z., Molnar T. (2018). Relationship between Cardiac Troponin and Thrombo-Inflammatory Molecules in Prediction of Outcome after Acute Ischemic Stroke. J. Stroke Cerebrovasc. Dis..

[15] Krings T., Willmes K., Becker R., Meister I.G., Hans F.J., Reinges M.H., Mull M., Thron A. (2006). Silent microemboli related to diagnostic cerebral angiography: A matter of operator’s experience and patient’s disease. Neuroradiology.

[16] Feriel J., Adamo F., Monneret D., Trehel-Tursis V., Favard S., Tsé C., Puybasset L., Bonnefont-Rousselot D., Imbert-Bismut F. (2015). S100B protein concentration measurement according to two different immunoassays. Clin. Chem. Lab. Med..

